# Supplementary material for: Evaluation of Porcine Gastric Mucin-Based Method for Extraction of Noroviruses from Seaweed Salad
Source: Viruses. 2025 Sep 16;17(9):1245. doi: 10.3390/v17091245 (PMC12474201; doi:10.3390/v17091245)
Supplement: Supplementary file 1 [file viruses-17-01245-s001.zip › viruses-3810676-supplementary.pdf]

Table S1. List of ingredients.

Brand A.

Seaweed, Black fungus, Sesame oil, String agar, White sesame seed, Sugar, Chili, White vinegar, Soy sauce (soybean, wheat, salt, water), Sorbitol, Salt, Monosodium glutamate, Citric acid, Tartrazine, Brilliant blue FCF.

Brand B

Seaweed, Sugar, High fructose corn syrup, Corn syrup, Agar-agar, Hydrolyzed vegetable protein (water, Corn, Soybeans, Wheat), Sesame seed oil, Distilled vinegar, Sesame seed, Black fungus, Salt, Chili pepper, Tartrazine, Brilliant Blue FCF.

Brand C

Seaweed, Seasoning (sugar, vinegar, sesame oil, disodium 5'-inositate, disodium 5'-guanyate, xanthan gum), Kikurage mushroom, Agar-agar, Salt, Sesame seed, Chili pepper, Tartrazine, Brillant blue, FCF

Brand D

Seaweed, Sugar, High fructose corn syrup, Corn syrup, Agar-agar, Hydrolyzed vegetable protein (water, Corn, Soybeans, Wheat), Sesame seed oil, Distilled vinegar, Sesame seed, Black fungus, Salt, Chili pepper, Tartrazine, xanthan gum, Brilliant Blue FCF.

Brand E

Seaweed, Black fungus, Sesame oil, String agar, White sesame seed, Sugar, Chili, White vinegar, Soy sauce (soybean, wheat, salt, water), Sorbitol, Salt, Monosodium glutamate, Citric acid, Tartrazine, Brilliant blue FCF.

Tables S2. Impact of the RNA extraction method

In the reference protocol from Suresh et al**.** (*Food Microbiology*, 84 (2019), 103254), RNA extraction from viruses captured by PGM-MB involved resuspending the beads in 50 µl of ultrapure water and heating at 100 °C for 10 minutes. In this study, two RNA extraction method, boiling at 100 °C for 10 minutes and the BOOM technology using the RNeasy kit, were compared for their efficiency in recovering HuNoV RNA. The recovery efficiency was assessed both from the original inoculum (Table S2a) and from viruses captured by PGM-MB without matrix (Table S2b).

a) Comparison of the HuNoV GII inoculum concentration by RT-qPCR following RNA extraction using boiling (10 min @ 100°C) or using the RNeasy kit.

Inoculum extraction	HuNoV GII<br>Inoculum average ± sd (gEq per 25 g)
10 min @ 100°C	432 ± 42
RNeasy	890 ± 110*

n=3, Inoculum volume tested was 50 µl. ANOVA **p*=0.03.

b) Impact of the RNA extraction process on the HuNoV GII recovery rates using the reference PGM-MB without matrix.

RNA extraction	HuNoV GII<br>PGM-MB Recovery rates (average ± sd)
10 min @ 100°C	32 ± 3
RNeasy	41 ± 2*

n=3, The inoculum concentration was estimated following its extraction using the RNeasy kit at 5.1 $\times 10^3$ gEq per 25g. The PGM-MB eluates volumes were in 50 µl. ANOVA **p*=0.013.

a)

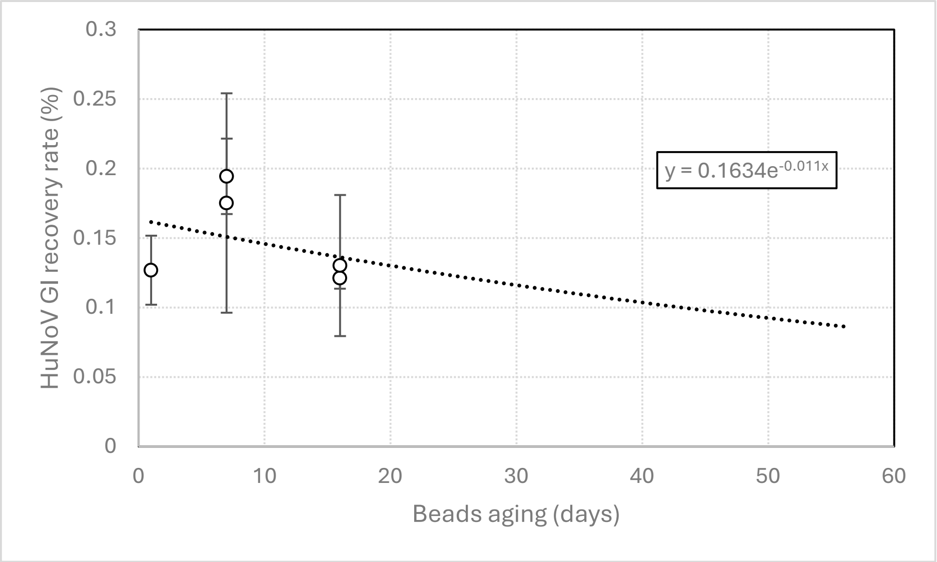

b)

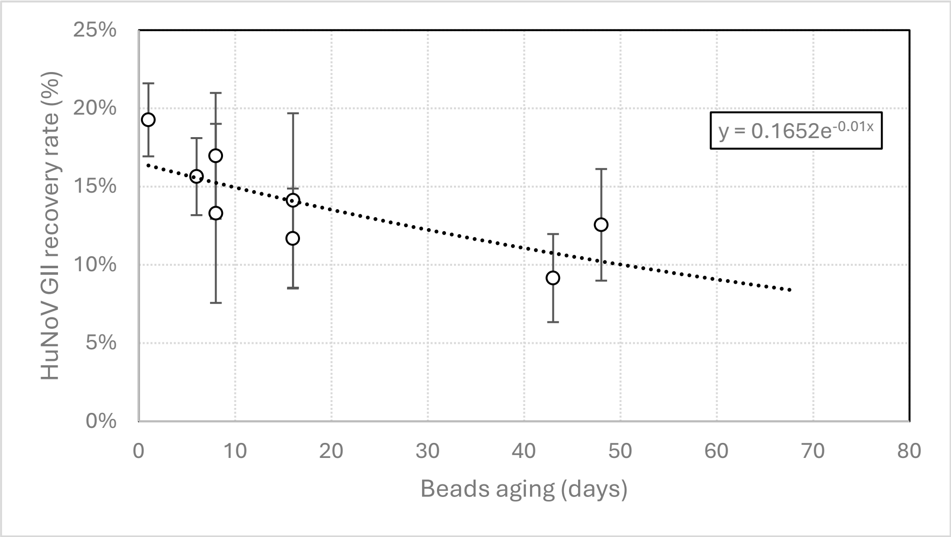

Figure S1. Impact of the PGM-MB preparation aging on optimized PGM-MB method recovery rates from Wakame seaweed salads. The HuNoV GI (a) and HuNoV GII (b) recovery rates were tested over several days. The average (○) and standard deviation (error bar) of triplicate assays and estimated exponential decay are represented.

a)

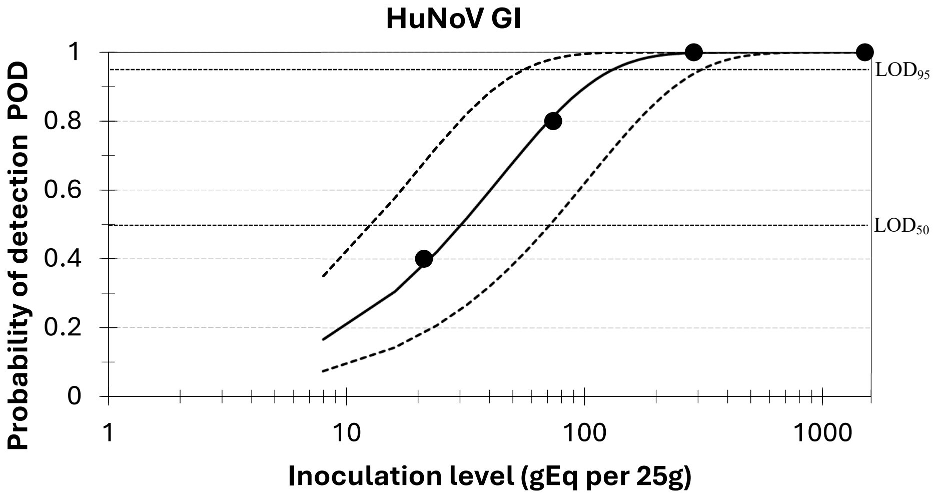

b)

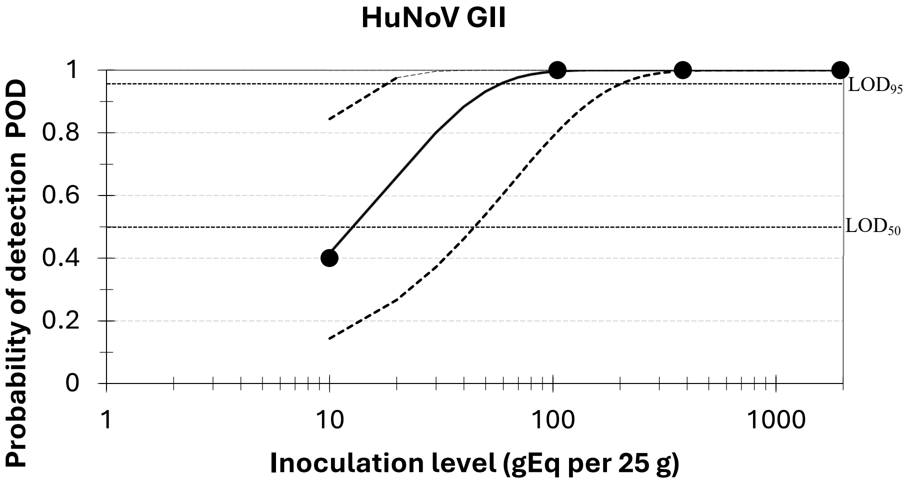

Figure S2. HuNoV LOD estimates from Wakame seaweed salad using the optimized PGM-MB extraction method. The proportion of positive observations for each concentration was used to assess the probability of detection and calculate the limit of detection (LOD_{50} and LOD_{95}) of HuNoV GI (a) and HuNoV GII (b). Upper and lower. POD 95% confidence bands are represented with dash. Each observed value represents three to five extractions.
